# HMOs Exert Marked Bifidogenic Effects on Children’s Gut Microbiota Ex Vivo, Due to Age-Related *Bifidobacterium* Species Composition

**DOI:** 10.3390/nu15071701

**Published:** 2023-03-30

**Authors:** Danica Bajic, Frank Wiens, Eva Wintergerst, Stef Deyaert, Aurélien Baudot, Pieter Van den Abbeele

**Affiliations:** 1 Glycom A/S-DSM Nutritional Products Ltd., Kogle Allé 4, 2970 Hørsholm, Denmark; 2 DSM Nutritional Products Ltd., Wurmisweg 576, 4303 Kaiseraugst, Switzerland; 3 Cryptobiotix SA, Technologiepark-Zwijnaarde 82, 9052 Ghent, Belgium

**Keywords:** HMOs, bifidobacteria, prebiotic, children, adult, gut microbiota, SCFA, aromatic lactic acids, GABA

## Abstract

Prebiotics are substrates that are selectively utilized by host microorganisms, thus conferring a health benefit. There is a growing awareness that interpersonal and age-dependent differences in gut microbiota composition impact prebiotic effects. Due to the interest in using human milk oligosaccharides (HMOs) beyond infancy, this study evaluated how HMOs [2’Fucosyllactose (2’FL), Lacto-N-neotetraose (LNnT), 3’Sialyllactose (3’SL), 6’Sialyllactose (6’SL)] and blends thereof affect the microbiota of 6-year-old children (*n* = 6) and adults (*n* = 6), compared to prebiotics inulin (IN) and fructooligosaccharides (FOS). The ex vivo SIFR^®^ technology was used, given its demonstrated predictivity in clinical findings. First, HMOs and HMO blends seemed to maintain a higher α-diversity compared to FOS/IN. Further, while 2′FL/LNnT were bifidogenic for both age groups, 3′SL/6′SL and FOS/IN were exclusively bifidogenic for children and adults, respectively. This originated from age-related differences in microbiota composition because while 3′SL/6′SL stimulated *B. pseudocatenulatum* (abundant in children), FOS/IN enhanced *B. adolescentis* (abundant in adults). Moreover, all treatments significantly increased acetate, propionate and butyrate (only in adults) with product- and age-dependent differences. Among the HMOs, 6′SL specifically stimulated propionate (linked to *Bacteroides fragilis* in children and *Phocaeicola massiliensis* in adults), while LNnT stimulated butyrate (linked to *Anaerobutyricum hallii* in adults). Indole-3-lactic acid and 3-phenyllactic acid (linked to immune health) and gamma-aminobutyric acid (linked to gut-brain axis) were most profoundly stimulated by 2′FL and HMO blends in both children and adults, correlating with specific *Bifidobacteriaceae*. Finally, 2′FL/LNnT increased melatonin in children, while 3′SL remarkably increased folic acid in adults. Overall, age-dependent differences in microbiota composition greatly impacted prebiotic outcomes, advocating for the development of age-specific nutritional supplements. HMOs were shown to be promising modulators in the adult, and particularly the children’s microbiota. The observed HMO-specific effects, likely originating from their structural heterogeneity, suggest that blends of different HMOs could maximize treatment effects.

## 1. Introduction

The gut microbiota is considered to be a vital organ with critical functions in sustaining human health [1]. Bacterial metabolites can influence physiological functions, from maintaining intestinal barrier integrity or local immunity to impacting gut-brain communication, adaptive immunity or appetite [2,3,4,5]. The pool of metabolites consists of, among others, short-chain fatty acids (SCFAs), branched-chain fatty acids (bCFAs), biogenic amines, and gases that are derived from carbohydrate, protein, and lipid breakdown [5,6]. Besides these well-studied compounds, new bioactive molecules are being discovered due to the more frequent application of metabolomics [7,8]. For example, Laursen et al. (2021) demonstrated that breastmilk-promoted *Bifidobacterium* spp. convert aromatic amino acids (tryptophan, phenylalanine, and tyrosine) into their respective aromatic lactic acids (indole-3-lactic acid, 3-phenyllactic acid, and 4-hydroxyphenyllactic acid) [9]. Interestingly, indole-3-lactic acid is associated with the activation of the aryl hydrocarbon receptor (AhR), which is key for controlling intestinal homeostasis and immune responses. This receptor is also considered a key regulator in the microbiome-gut-brain axis, with AhR-activating microbial metabolites modulating multiple neuronal responses [10]. Another bacterial metabolite is the gamma-aminobutyric acid (GABA), a chief inhibitory neurotransmitter, which can also be produced by *Bifidobacterium* spp. [11,12]. GABA plays a pivotal role in anxiety and depression disorders, regulates blood pressure and heart rate, as well as increases expression of MUC1 that prevents bacterial invasion [13]. While the current knowledge is expanding, questions on how to manage the production of such health-related metabolites remain largely to be elucidated.

**Figure 1:**
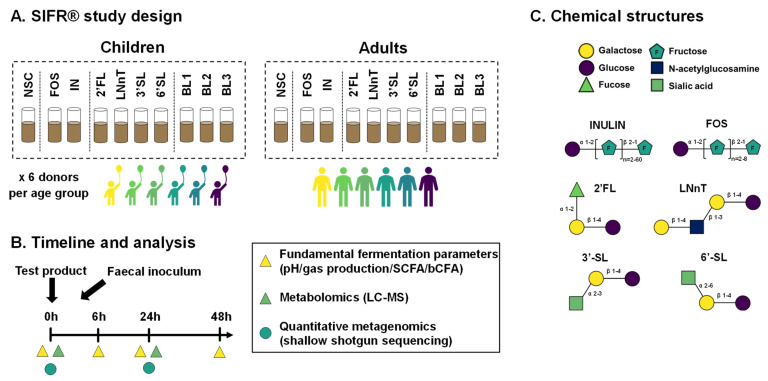
**Experimental design to assess prebiotic effects on the gut microbiota of two age groups (children and adults) ex vivo.** (**A**) Reactor design using the ex vivo SIFR^®^ technology to test the impact of single HMOs (2′FL, LNnT, 3′SL, 6′SL), mixtures thereof (BL1, BL2, BL3) and reference prebiotics (FOS and IN) on the gut microbiota of 6-year-old children and human adults, compared to a no substrate control (NSC). (**B**) Timeline and analysis at different time points. (**C**) Chemical structure of the test products. 2′FL = 2′Fucosyllactose; LNnT = Lacto-N-neotetraose; 3′SL = 3′Sialyllactose; 6′SL = 6′Sialyllactose; BL1/2/3 = HMO Blend 1/2/3; IN = inulin; FOS = fructooligosaccharides; SCFA = short-chain fatty acids; bCFA = branched fatty acids; LC-MS = Liquid chromatography coupled with mass spectrometry.

Strategies to impact the human gut microbiota often involve the use of live microorganisms, prebiotics, or combinations thereof. Prebiotics are defined as substrates that are selectively utilized by host microorganisms, conferring a health benefit [14]. Inulin-type fructans, including fructooligosaccharides (FOS) and inulin (IN), are natural components present in several fruits and vegetables, including chicory, onion, banana, garlic, artichoke, and many others [15]. IN and FOS are linear polymers of β-2,1-linked fructose monomers with a terminal glucose residue (degree of polymerization = 2–60 (IN) or 2–8 (FOS)) (Figure 1C). Human milk oligosaccharides (HMOs) are a more recent class of prebiotics that have increasingly been studied over the past decades. In contrast to fructans, HMOs are usually consumed in large amounts only by breastfed infants via human milk. The most abundant HMOs in human milk are 2′-fucosyllactose (2′-FL) and lacto-N-tetraose (LNnT) [16,17], with other HMOs, such as sialylated HMOs which are present in lower concentration but remain functional [18,19]. Compared to fructans, HMOs are smaller structures, consisting of a broader range of monosaccharide components and glycosidic linkage types, that define their accessibility as substrate for gut microbes (Figure 1C). Intervention studies in infants demonstrated that 2′FL and LNnT increase the abundance of *Bifidobacterium*, reduce *Escherichia* and *Peptostreptococcaceae* levels, while lowering the use of antibiotics beyond six months of intervention [20] is linked to the aforementioned antipathogenic effects. HMOs in children and adults are not extensively studied. Nevertheless, recent in vivo and in vitro studies revealed that HMOs have potential effects beyond infancy, given the observation of bifidogenic effects on gut barrier integrity and immunity [21,22], and antipathogenic effects against *Clostridioides difficile* [23]. Preliminary findings also reveal beneficial effects on the microbiota of irritable bowel syndrome patients [24]. When applied as a novel type of prebiotic for children or adults, dietary HMOs may induce beneficial microbiota-mediated health effects that augment effects of inulin-type fructans received with a normal diet.

A key contributing factor to the benefit of prebiotics, and HMOs in particular, is the microbiota by which they are fermented when reaching the colon. Interpersonal differences in microbiota composition among human adults are vast [25] and have been shown to impact the outcomes of interventions [26,27]. Besides interpersonal differences, the gut microbiota also evolves within an individual over time. With respect to *Bifidobacterium* species, it has been shown that *B. longum* subsp. *infantis*, *B. breve*, and *B. bifidum* are the dominant members during infancy [28]. Then, with the introduction of solid foods, a distinct *Bifidobacterium longum* clade expands [29]. Further, while the microbiota of healthy children (6–9 years old) is drastically enriched with *Bifidobacterium catenulatum* and *B. pseudocatenulatum* [30], the adult microbiota is enriched with *B. longum* and *B. adolescentis* [31]. When aiming to use prebiotics across age groups, one should thus consider such age-dependent differences in gut microbiota composition, along with interpersonal differences, as such differences could strongly affect the outcome of interventions.

In vitro gut models have the potential to complement human studies as they allow to reduce confounding factors, such as dietary patterns and transit time [32], that limit our understanding of mechanisms of action in vivo. A key advantage of in vitro gut models is that they allow to obtain insights into the bacterial metabolite that are rapidly absorbed in vivo [33]. Though, the current generation of in vitro models are confounded by drastic alterations between the in vivo-derived microbiota and the microbiota that eventually colonizes the lab systems, both for short-term [34,35,36,37] and long-term models [38,39]. Even the most sophisticated models result in a marked change in microbiota composition within only three days after inoculation [40]. This limits the ability of gut models to address interpersonal and age-dependent differences. The low throughput of the current generation of gut models is another technical limitation that obstructs the addressing of such differences. 

This study aimed to assess the impact of single HMOs (2′FL, LNnT, 3′SL and 6′SL) and blends thereof compared to inulin-type fructans (FOS and IN) on metabolite production and microbial composition of both children (6-year-olds) and adults. Fructans were selected as reference compounds, given its presence in a normal diet (as part of several fruits and vegetables) and its common use as dietary supplements. The research question was addressed using the ex vivo SIFR^®^ technology (systemic intestinal fermentation research), a recently validated bioreactor-based gut model [41]. Key features of this model are a sustained similarity between the microbiota in the reactors and the original in vivo samples (hence classifying as ex vivo model), along with a high throughput, technical reproducibility and importantly, a demonstrated predictivity: ex vivo findings within 24–48 h are predictive for outcomes of clinical studies where prebiotics are repeatedly dosed over 2–6 weeks. By testing the various products for 6 individuals per age group, insights into both interpersonal and age-dependent differences of treatment effects were obtained. 

## 2. Materials and Methods

### 2.1. Test Products

Four HMOs (2′Fucosyllactose (2′FL): DSM GlyCare^TM^ 2FL 9000, Lacto-N-neotetraose (LNnT): DSM GlyCare^TM^ LNnT 9000, 3′Sialyllactose (3′SL): DSM GlyCare^TM^ 3SL 9001, 6′Sialyllactose (6′SL): DSM GlyCare^TM^ 6SL 9001) and three HMO blends (BL1 = 77.86% 2′FL; 6.49% 3′SL; 15.65% 6′SL (% *w*/*w*); BL2 = 87.55% 2′FL; 12.45% LNnT (% *w*/*w*); BL3 = 70.1% 2′FL; 9.97% LNnT; 5.84% 3′SL; 14.09% 6′SL (% *w*/*w*)) were compared to two reference products (inulin from chicory (IN): Sigma I2255, and fructooligosaccharides (FOS) from chicory: Sigma F8052). All products were tested at a dose equivalent to 5 g/day and compared to a no substrate control (NSC), in which the microbial communities were grown in the absence of additional test products, yet in the presence of an optimized nutritional medium.

### 2.2. SIFR^®^ Technology

The SIFR^®^ technology was recently validated and it enables to study the human gut microbiota in a highly biorelevant manner for multiple test conditions (both treatments and test subjects) [41]. Individual bioreactors were briefly processed in parallel to a bioreactor management device (Cryptobiotix, Ghent, Belgium). Each bioreactor contained 5 mL of nutritional medium-fecal inoculum blend supplemented with test products, then sealed individually, before being rendered anaerobic. Blend M0003 was used to prepare the nutritional medium (Cryptobiotix, Ghent, Belgium). After preparation, bioreactors were incubated under continuous agitation (140 rpm) at 37 °C for 48 h (MaxQ 6000, Thermo Scientific, Thermo Fisher Scientific, Merelbeke, Belgium). Upon gas pressure measurement in the headspace, liquid samples were collected for subsequent analysis. Fresh fecal samples were collected according to a procedure approved by Ethics Committee of the University Hospital Ghent (reference number BC-09977). This involved the human adults or parents (of the 6-year-old children) signing an informed consent in which they donate their fecal sample or one of their child’s samples for the current study. The selection criteria for the human adult donors were: no antibiotic use in the past 3 months, no gastrointestinal disorders (cancer, ulcers, IBD), no use of probiotic, non-smoking, alcohol consumption < 3 units/d and BMI < 30. For this specific study, four male and two female human adults were tested with an average age of 30 (±4 years). Further, three male and three female 6-year-old children were included. 

### 2.3. Experimental Design, Timeline and Analysis

Ten study arms were tested for each of the twelve fecal microbiota: (i) NSC containing background medium and fecal microbiota without products, (ii) reference prebiotics (FOS and IN), (iii) single HMOs (2′FL, LNnT, 3′SL and 3′SL), and (iv) 3 HMO blends (BL1, BL2 and BL3) (Figure 1). Samples were collected at 0 h (NSC only), 6 h, 24 h and 48 h for fundamental fermentation parameters (pH, gas production, short-chain fatty acids (SCFA) and branched-chain fatty acids (bCFA)). Samples at 0 h (NSC only) and 24 h were collected for community composition analysis (quantitative shotgun sequencing) and metabolomics (LC-MS) (Figure 1). 

### 2.4. Fundamental Fermentation Parameters

SCFA (acetate, propionate, butyrate and valerate) and bCFA (sum of isobutyrate, isocaproate and isovalerate) were determined via GC with flame ionization detection (Trace 1300, Thermo Fisher Scientific, Merelbeke, Belgium), upon diethyl ether extraction as previously described [42]. pH was measured using an electrode (Hannah Instruments Edge HI2002, Temse, Belgium).

### 2.5. Microbiota Phylogenetic Analysis via Quantitative Shallow Shotgun Sequencing 

Quantitative data was obtained by correcting abundances (%; shallow shotgun sequencing) with total cell counts for each sample (cells/mL; flow cytometry), resulting in estimated cell counts/mL of different taxonomic groups.

Initially, a bacterial cell pellet was obtained by centrifugation of 1 mL sample for 5 min at 9000× *g*. DNA was extracted via the SPINeasy DNA Kit for Soil (MP Biomedicals, Eschwege, Germany), according to the manufacturer’s instructions. Subsequently, DNA libraries were prepared using the Nextera XT DNA Library Preparation Kit (Illumina, San Diego, CA, USA) and IDT Unique Dual Indexes with a total DNA input of 1 ng. Genomic DNA was fragmented using a proportional amount of Illumina Nextera XT fragmentation enzyme. Unique dual indexes were added to each sample followed by twelve cycles of PCR to construct libraries. DNA libraries were purified using AMpure magnetic Beads (Beckman Coulter, Brea, CA, USA), eluted in QIAGEN EB buffer, quantified using a Qubit 4 fluorometer and a Qubit dsDNA HS Assay Kit, and sequenced on an Illumina Nextseq 2000 platform 2 × 150 bp. Unassembled sequencing reads were converted to relative abundances (%) using the CosmosID-HUB Microbiome Platform (CosmosID Inc., Germantown, MD, USA) [43,44]. 

For total cell count analysis, liquid samples were diluted in anaerobic phosphate-buffered saline (PBS), after which cells were stained with SYTO 16 at a final concentration of 1 µM and counted via a BD FACS Verse flow cytometer (BD, Erembodegem, Belgium). Data was analyzed using FlowJo, version 10.8.1.

### 2.6. Metabolomic Analysis

A liquid chromatography–mass spectrometry (LC-MS) analysis was carried out using a Thermo Scientific Vanquish LC coupled with Thermo Q Exactive HF MS (Thermo Scientific, Belgium). An electrospray ionization interface was used as the ionization source. Analysis was performed in negative and positive ionization mode. The UPLC was performed using a slightly modified version of the protocol described by Doneanu et al. 2011 [45]. Peak areas were extracted using Compound Discoverer 3.1 (Thermo Scientific). In addition to the automatic compound extraction by Compound Discoverer 3.1, a manual extraction of compounds included in an in-house library, was performed using Skyline 21.1 (MacCoss Lab Software) [46]. Identification of compounds were performed at three levels: level (1) identification by retention times (compared against in-house authentic standards), accurate mass (with an accepted deviation of 3 ppm), and MS/MS spectra; level (2a) identification by retention times (compared against in-house authentic standards), accurate mass (with an accepted deviation of 3 ppm); level (2b) identification by accurate mass (with an accepted deviation of 3 ppm), and MS/MS spectra; level (3) identification by accurate mass alone (with an accepted deviation of 3 ppm).

A quality control of the technical variability was performed by analysing a QC sample, which considered a pooled sample of all samples. This QC sample was run after every six samples to monitor instrument performance. Following the PCA based on the individual values of level 1 annotated metabolites, QC samples were grouped closely together, indicating that the biological variance (treatment effects and interpersonal differences across donors) exceeded the analytical variance (Figure S1A,B). 

Children and adult datasets were analyzed independently. A selection of 75 metabolites for each age group belonging to level ½ and previously linked to the gut microbiome were further evaluated. Out of these 75 metabolites, the levels of 39 (adults) or 46 (children) metabolites increased along the 24 h incubations (for at least four of the six donors) and 33 (adults) or 30 (children) were significantly affected (FDR = 0.1). These subsets of metabolites were considered for further statistical analysis and graphical representation.

### 2.7. Data Analyses

All univariate and multivariate analyses were performed by GraphPad Prism (v9.3.1; www.graphpad.com; accessed on 26 December 2022), while Regularized Canonical Correlation Analysis (rCCA) was executed using the mixOmics package with the shrinkage method for estimation of penolization parameters (version 6.16.3) in R (4.1.1; www.r-project.org; accessed on 26 December 2022) [47]. Treatment effects were compared with the NSC using repeated-measures ANOVA analysis (based on paired testing) and *p*-values were corrected with the Benjamini–Hochberg method [48] (FDR = 0.05 or 0.10 as indicated). Paired testing (repeated-measures ANOVA) was performed for setups considering six donors in *n* = 1. For analysis of microbial composition, three measures were taken. First, aforementioned statistical analysis was performed on the log_10_-transformed values. Second, a value of a given taxonomic group below the limit of detection (LOD) was considered equal to the overall LOD, according to the procedure elaborated by Van den Abbeele et al. (2023) [41]. Finally, a threshold was set in order to retain the 100 most abundant species in the analysis, to avoid excessive *p*-values corrections.

## 3. Results

### 3.1. Age-Dependent Fecal Microbial Community Composition 

Fecal microbiota composition of children and adults was significantly different (*p* = 0.024 based on Bray-Curtis distance) (Figure S2A). While the children’s microbiota was enriched with *Bifidobacterium catenulatum*, *Bifidobacterium pseudocatenulatum*, and *Phocaeicola vulgatus*, amongst others, the adult microbiota was enriched with *Bifidobacterium adolescentis* and *Phocaeicola massiliensis,* among other taxa (Figure S1B)*. Bifidobacteriaceae* were thus a key taxonomic group to differentiate between both age groups. Further, the different composition between children and adults provides a rationale for assessing the impact of HMOs in both groups.

### 3.2. Kinetic Sampling Allowed to Cover Saccharolytic and Proteolytic Fermentation Processes

**Figure 2:**
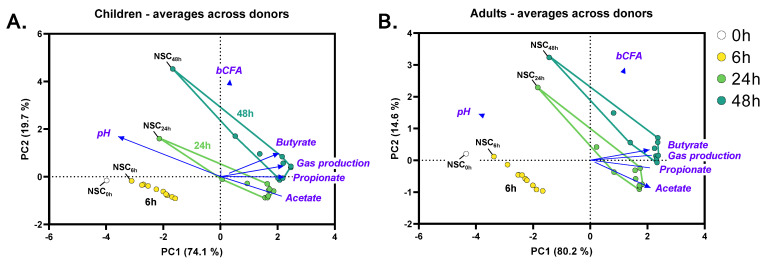
**Kinetic sampling covered saccharolytic (0–24 h) and proteolytic fermentation processes (24–48 h).** Principal component analysis (PCA) summarizing the levels of fundamental fermentation parameters (pH, SCFA, bCFA and gas production), as averaged across 6 children (**A**) or 6 human adults (**B**) at different time points (0 h, 6 h, 24 h, and 48 h) in the no substrate control (NSC) or upon treatment with reference prebiotics (FOS/IN), single HMOs or HMOs blends. SCFA—short-chain fatty acids; bCFA—branched fatty acids; IN—inulin; FOS—fructooligosaccharides; HMO—human milk oligosaccharide.

The gut microbiota of children and adults was metabolically active along the 48 h incubations, both in the NSC and upon treatment (FOS/IN/HMOs/HMO blends), as illustrated by PCA plots based on fundamental fermentation parameters (Figure 2). As time progressed, samples moved to the right side of the PCA, suggesting enhanced production of SCFA. At each time point, the NSC positioned closest to the 0 h samples, suggesting that the treatments boosted metabolite production compared to the NSC.

The kinetic sampling (0 h, 6 h, 24 h and 48 h) grasped different stages of the fermentation processes. While information on the initial speed of fermentation was obtained at 6 h, most SCFA production from saccharolytic fermentation occurred between 0 h and 24 h (as illustrated for 2′FL in Figure S3C,D). Further, 48 h samples moved upwards along PC2 as opposed to 24 h samples (Figure 2), reflecting an additional bCFA production derived from the fermentation of aromatic amino acids between 24 and 48 h (Figure S3A,B). Overall, this suggests that saccharolytic fermentation mainly occurred between 0 and 24 h, with additional proteolytic activity between 24 and 48 h. One could thus compare the observations in aforementioned time frames with the observations along the proximal (0–24 h) and distal colon (24–48 h). Based on these first insights, the 24 h time point was selected for the in-depth analysis of prebiotic effects on microbial composition and metabolite production.

### 3.3. HMOs and HMO Blends Maintained a Higher α-Diversity Compared to Fructans, Especially for Adults

**Figure 3:**
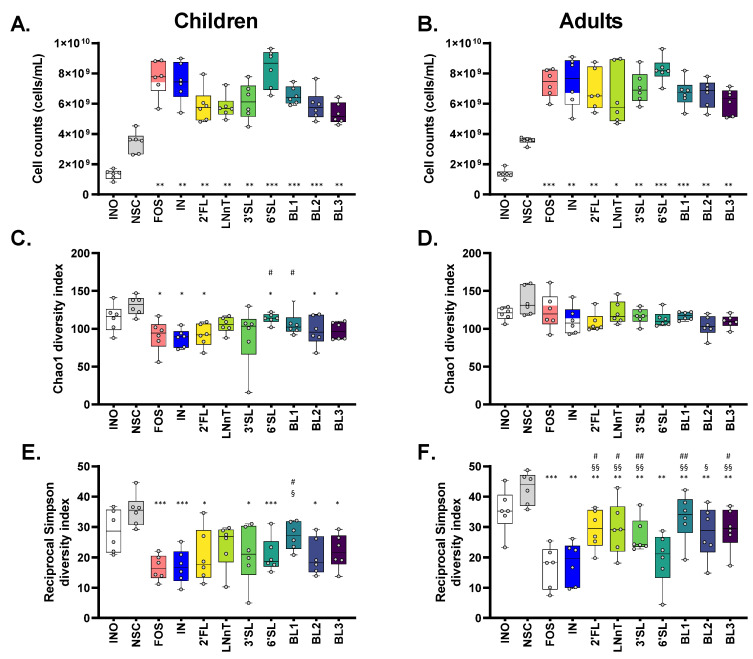
**All treatments increased bacterial cell density, with HMOs and HMO blends maintaining a higher α-diversity compared to fructans.** Impact of single HMOs (2′FL, LNnT, 3′SL, 6′SL), mixtures thereof (BL1, BL2, BL3) and reference prebiotics (FOS and IN) on cell density (**A**,**B**) and α-diversity (**C**–**F**) of the gut microbiota of 6-year-old children and adults (*n* = 6), at 24 h upon initiation of treatment, compared to a no substrate control (NSC), as tested with the ex vivo SIFR^®^ technology. Statistical differences between treatments and NSC are indicated with asterisks [* (*p*_adjusted_ < 0.05), ** (*p*_adjusted_ < 0.01) or *** (*p*_adjusted_ < 0.001)]. Further, statistical differences between single HMOs/HMO blends and FOS are indicated with §/§§/§§§, while differences with IN are indicated with #/##/###. 2′FL —2′Fucosyllactose; LNnT—Lacto-N-neotetraose; 3′SL—3′Sialyllactose; 6′SL—6′Sialyllactose; BL1/2/3 = HMO Blend 1/2/3; IN—inulin; FOS—fructooligosaccharides.

The marked metabolite production coincided with a markedly increased cell density (Figure 3A,B). All products increased cell density from 3 × 10^9^ cells/mL in the NSC up to 5–10 × 10^10^ cells/mL (Figure 3A,B). Due to the large differences in cell densities between test conditions, it was critical to convert proportional outcomes of shotgun sequencing to absolute levels (Figure S4). Subsequent data processing were based on quantitative outcomes.

Despite the marked growth in the NSC (factor 2–3 increase), microbial diversity—both in terms of richness (Chao1 diversity index) and evenness (reciprocal Simpson diversity index)—was maintained in the NSC (Figure 3C–F), suggesting that the SIFR^®^ technology supported the growth of a complete spectrum of in vivo-derived gut microbes.

Overall, single HMOs and HMO blends maintained a higher α-diversity than fructans, especially when supplied to the adult microbiota. First, all treatments (except for LNnT and 3′SL) reduced species richness compared to the NSC when supplied to the children’s microbiota, with 6′SL and BL1 maintaining a higher diversity compared to IN (Figure 3C). Species evenness tended to be higher for single HMOs and HMO blends compared to both fructans, although not reaching significance, given marked interpersonal differences (Figure 3D). For adults, species richness was similar for all treatments (Figure 3E), while diversity in terms of species evenness was significantly higher for single HMOs/HMO blends compared to the fructans, except for 6′SL and BL2 (Figure 3F). 

### 3.4. Children: HMOs Exert a Remarkable Bifidogenic Effect Compared to Fructans

**Figure 4:**
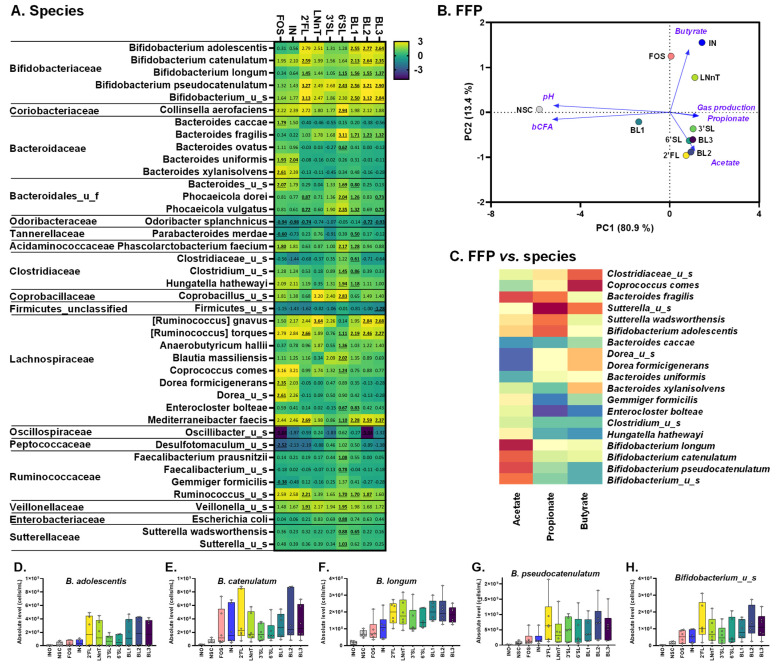
**HMOs exerted a remarkable effect on *Bifidobacterium* species when supplied to the children’s microbiota, in contrast to fructans.** Impact of single HMOs (2′FL, LNnT, 3′SL, 6′SL), mixtures thereof (BL1, BL2, BL3) and reference prebiotics (FOS and IN) on microbial composition at species level at 24 h upon initiation of treatment, as tested with the ex vivo SIFR^®^ technology for 6-year-old children. (**A**) Heatmap representing average values of microbial taxa (*n* = 6) that were significantly affected by any of the treatments (FDR = 0.10), expressed as log2(ratio treatment vs. NSC). Significant differences are indicated by bold and underlining. (**B**) PCA summarizing treatment effects on fundamental fermentation parameters, based on the average data across donors (*n* = 6). (**C**) Regularized Canonical Correlation Analysis (rCCA) (cut off = 0.65) to highlight correlations between short-chain fatty acids (acetate, propionate, butyrate) and significantly affected species. (**D**–**H**) Box plots representing the abundances (cells/mL) of different *Bifidobacterium* species. INO = inoculum; NSC = no substrate control; 2′FL = 2′Fucosyllactose; LNnT = Lacto-N-neotetraose; 3′SL = 3′Sialyllactose; 6′SL = 6′Sialyllactose; BL1/2/3 = HMO Blend 1/2/3; IN = inulin; FOS = fructooligosaccharides.

When administered to the children’s microbiota, all treatments significantly increased acetate and propionate compared to NSC (Figure S5A,C). Single HMOs and HMO blends additionally increased acetate compared to fructans. While none of the treatments significantly increased butyrate, given large interpersonal differences, FOS, IN and LNnT mostly increased butyrate (Figure S4E and Figure 4B). Similarly, 6′SL mostly increased propionate (Figure S4C). 

Unlike fructans (FOS/IN), single HMOs and HMO blends significantly increased *Bifidobacteriaceae* (Figure S6A,C) due to the increase in the five *Bifidobacterium* species: *B. longum, B. adolescentis*, *Bifidobacterium*_u_s and especially *B. pseudocatenulatum*, and *B. catenulatum* (Figure 4A,D–H). The involvement of particularly, *B. pseudocatenulatum, B. catenulatum* and *B. longum,* as key HMO-fermenting species followed from their marked correlation with acetate (Figure 4C), which is the main metabolite of *Bifidobacterium* spp. [49]. Besides exerting bifidogenic effects, HMOs also stimulated other microbial species. 2′FL increased *Ruminococcus torques* and *Mediterraneibacter faecis*, while 6′SL exerted a remarkable effect on *Bacteroides fragilis*, among others, that strongly related with acetate/propionate. LNnT markedly increased *Ruminocococus gnavus*, and interestingly also tended to increase the buytrate-producing *Coprococcus comes* and *Anaerobutyricum hallii*. 

**Figure 5:**
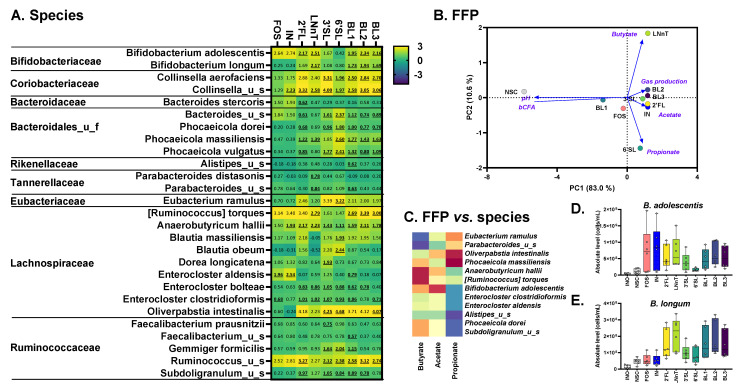
**Fructans, 2′FL and LNnT exerted bifidogenic effects when supplied to the adult microbiota, in contrast to sialylated HMOs that rather increased *Bacteroidaceae*.** Impact of single HMOs (2′FL, LNnT, 3′SL, 6′SL), mixtures thereof (BL1, BL2, BL3) and reference prebiotics (FOS and IN) on microbial composition at species level at 24 h upon initiation of treatment, as tested with the ex vivo SIFR^®^ technology for human adults. (**A**) Heatmap representing average values of microbial taxa (*n* = 6) that were significantly affected by any of the treatments (FDR = 0.10), expressed as log2(ratio treatment vs. NSC). Significant differences are indicated by bold and underlining. (**B**) PCA summarizing treatment effects on fundamental fermentation parameters, based on the average data across donors (*n* = 6). (**C**) Regularized Canonical Correlation Analysis (rCCA) (cut off = 0.65) to highlight correlations between short-chain fatty acids (acetate, propionate, butyrate) and significantly affected species. (**D**,**E**) Box plots representing the abundances (cells/mL) of different *Bifidobacterium* species. INO—inoculum; NSC—no substrate control; 2′FL—2′Fucosyllactose; LNnT—Lacto-N-neotetraose; 3′SL—3′Sialyllactose; 6′SL—6′Sialyllactose; BL1/2/3—HMO Blend 1/2/3; IN—inulin; FOS—fructooligosaccharides.

In contrast to the HMOs, fructans strongly increased *Bacteroidaceae*, *Lachnospiraceae*, and *Acidaminococcaceae* (Figure S6B,D). At lower taxonomic levels, FOS and IN stimulated *Bacteroides uniformis*, while FOS also increased the levels of *Bacteroides caccae*, *Bacteroides xylanisolvens*, *Bacteroides*_u_s, *Phascolarctobacterium faecium*, *Phocaeicola dorei*, *Phocaeicola vulgatus*, and two *Dorea* spp (Figure 5A). Both fructans specifically increased *C. comes* that markedly correlated with butyrate that was indeed increased for fructans.

### 3.5. Adults: 2′FL/LNnT Exert Bifidogenic Effects unlike 3′SL/6′SL That Boost Bacteroidaceae

When administered to the adult microbiota, all treatments significantly increased acetate, propionate, and also butyrate, in contrast to the children’s microbiota. (Figure S5B,D). LNnT and 6′SL exerted remarkable effects on butyrate and propionate, respectively (Figure S4D,F and Figure 5B). 

All treatments, except 3′SL and 6′SL, significantly increased *Bifidobacteriaceae* (Figure S6B) due to the increase in *B. adolescentis* and *B. longum* (Figure 5A,D,E), with *B. longum* specifically increasing for 2′FL and LNnT. A remarkable finding for *B. adolescentis* was its strong correlation with acetate, suggesting it is a key 2′FL/LNnT/fructan-fermenting species. Besides exerting bifidogenic effects, 2′FL and LNnT exerted a profound effect on *Anaerobutyricum hallii* and *Ruminococcus torques* as both strongly related with butyrate levels. Interestingly, LNnT most strongly increased *A. hallii* potentially explaining the marked effect of LNnT on butyrate.

The sialylated HMOs impacted the adult microbiota very differently compared to 2′FL and LNnT. In contrast, when dosed to the children’s microbiota, sialylated HMOs did not induce bifidogenic effects. 3′SL and 6′SL, such as fructans, significantly increased *Bacteroidaceae* due to the increase in a spectrum of species (*Phocaeicola dorei*, *Phocaeicola massiliensis* and *Phocaeicola vulgatus).* Moreover, along with other effects, 3′SL and 6′SL specifically increased *Eubacterium ramulus* and *Gemmiger formicilis.* Finally, all HMOs exerted what seemed to be HMO-specific effects on *Phocaeicola massiliensis*, *Oliverpabstia intestinalis* (both strongly correlating with propionate levels) along with *Coriobacteriaceae* (*Collinsella* spp.).

### 3.6. A Spectrum of Health-Related Metabolites Increased (Aromatic Lactic Acids, HICA and GABA)

The metabolomics analysis revealed product-specific effects that were relatively consistent across all age groups. As elaborated below, *Bifidobacterium*-related metabolites were more profoundly affected upon administration to the children’s microbiota.

**Figure 6:**
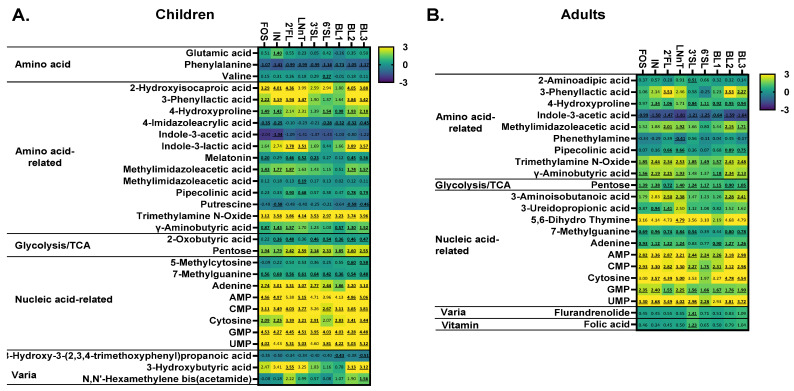
**A spectrum of health-related metabolites increased (aromatic lactic acids, HICA and GABA).** Impact of single HMOs (2′FL, LNnT, 3′SL, 6′SL), mixtures thereof (BL1, BL2, BL3) and reference prebiotics (FOS and IN) on gut metabolomics signatures in children (**A**) and adults (**B**) at 24 h upon initiation of treatment, as tested with the ex vivo SIFR^®^ technology. Heatmap representing average values of subsets of metabolites (*n* = 6) that were significantly affected by any of the treatments (FDR = 0.10), expressed as log2(ratio treatment vs. NSC). Significant differences are indicated by bold and underlining. 2′FL = 2′Fucosyllactose; LNnT = Lacto-N-neotetraose; 3′SL = 3′Sialyllactose; 6′SL = 6′Sialyllactose; BL1/2/3 = HMO Blend 1/2/3; IN = inulin; FOS = fructooligosaccharides.

For children, 3-phenyllactic acid, an aromatic lactic acid derived from phenylalanine (that was efficiently consumed; data not shown) increased significantly for all treatments, except sialylated HMOs and BL1 (Figure 6A). A similar observation was conducted for 2-hydroxyisocaproic acid (HICA), a leucine metabolite. Further, indole-3-lactic acid, another aromatic lactic acid (derived from tryptophan), increased significantly for 2′FL, LNnT, BL2, and BL3. Interestingly, *Bifidobacteriaceae* (*Bifidobacterium* spp.), *Lachnospiraceae (R. torques, M. faecis*), and *Ruminococacceae* (*Ruminococcus*_u_s) strongly correlated with the HICA, indole-3-lactic acid, and 3-phenyllactic acid (Figure S7). The γ-aminobutyric acid (GABA), a glutamate metabolite, increased for all test conditions, reaching significance for fructans, 2′FL, and the three HMO blends. A marked correlation between GABA and *Bifidobacterium* spp. was established, while *Bacteroides/Phocaeicola* species correlated with GABA for fructans (Figure S7). Another metabolite related to the gut-brain axis, 3-hydroxybutyric acid, increased for all treatments, reaching significance for 2′FL, BL2, and BL3. 

For adults, milder effects were observed for the 3-phenyllactic acid as compared to children, with significant increases being noted for 2′FL, BL2 and BL3 (Figure 6B), correlating with the presence of two *Bifidobacterium* spp. (*B. adolescentis, B. longum*), *R. torques*, *A. hallii*, and *Ruminococcus*_u_s (Figure S8). Next, GABA increased significantly for all test products, except for sialylated HMOs. Upon 2′FL supplementation, GABA correlated with *B. adolescentis*, while for 3′SL/6′SL, correlations with *P. vulgatus* were noted (Figure S8). Finally, folic acid increased specifically upon 3′SL supplementation. Interestingly, folic acid levels markedly correlated with *O. intestinalis* (Figure S8). 

## 4. Discussion

This study evaluated the effect of single HMOs and HMO blends on the children’s and adult gut microbiota. The modulatory effect of HMOs was weighted against well-studied fructans (IN and FOS). The research question was addressed using the ex vivo SIFR^®^ technology, a novel technology that provides insights into gut microbiota modulation that are predictive for observations of repeated intake clinical studies (down to species level resolution) [41]. Moreover, a key feature of the SIFR^®^ technology is the sustained similarity between the original donor microbiota and the microbiota growing in the SIFR^®^ reactors, classifying the technology as an ex vivo technology. This was critical for our study, given the focus on age-dependent differences in microbiota composition that had to be preserved along the entire duration of the ex vivo experiment. An example of such key age-dependent difference was in line with recent in vivo findings [30,31], *B. pseudocatenulatum* and *B. catenulatum* were abundant in children, whereas *B. adolescentis* was abundant in adults. These age-dependent differences were preserved along the SIFR^®^ experiments and greatly affected the impact of HMOs and fructans on gut microbiota composition and metabolite production effects.

While 2′FL/LNnT were bifidogenic for both age groups, 3′SL/6′SL and FOS/IN were exclusively bifidogenic for children and adults, respectively. This remarkable age-dependent treatment response was linked with the aforementioned age-dependent differences in microbiota composition: whereas the main *Bifidobacterium* species of children—*B. pseudocatenulatum*—was strongly stimulated by 3′SL/6′SL, the main species for adults—*B. adolescentis*—was enhanced by FOS/IN, confirming earlier findings [50,51]. The strong involvement of *Bifidobacterium* species as key contributors to the fermentation of the test products was stressed by the marked correlation of *Bifidobacterium* species with acetate, which is indeed the main metabolite of *Bifidobacterium* spp. [49]. For children, *B. pseudocatenulatum, B. catenulatum* and *B. longum* markedly correlated with acetate, suggesting they are the key HMO-fermenting species for children, while for adults, a remarkable correlation with *B. adolescentis* was established, suggesting the involvment of *B. adolescentis* in 2′FL/LNnT/fructan fermentation for adults. It is to be noted that since HMO utilization is shown to be dependent on the type of HMO and the exact *Bifidobacterium* strain [52], there thus seems to be a certain degree of consistency in HMO utilization among strains of a specific species associated with a given age group. Overall, given the distinct health-related properties of *Bifidobacteriaceae* [28,31,53], health benefits can be expected from the administration of all tested HMOs to children and 2′FL/LNnT/fructans to adults.

This health-related potential of bifidogenic effects was stressed upon applying a metabolomics approach during the current study, revealing that besides acetate, a spectrum of other health-related *Bifidobacterium*-mediated metaboltes strongly increased particularly for 2′FL and BL2/3, including aromatic lactic acids (indole-3-lactic acid, 3-phenlyllactic acid), HICA, GABA and melatonin. Interestingly, Laursen et al. (2021) recently reported that breastmilk-promoted *Bifidobacterium* spp. can indeed convert aromatic amino acids (tryptophan, phenylalanine, and tyrosine) into their respective aromatic lactic acids (indole-3-lactic acid, 3-phenyllactic acid, and 4-hydroxyphenyllactic acid) via a previously unrecognized aromatic lactate dehydrogenase (ALDH) [9]. Indole-3-lactic acid, a ligand for the aryl hydrocarbon receptor (AhR), was measured in the forebrain of the mice [54]. AhR is a transcription factor expressed throughout the brain including on neurons, astrocytes, and endothelial cells forming the blood-brain-barrier (BBB) [55]. The activation of this transcription factor alters the innate and adaptive immune responses, while activation of its signaling in astrocytes limits CNS inflammation [56]. In vivo, AhR participates in hippocampal neurogenesis, and AhR-deficient mice displayed impaired hippocampal-dependent contextual fear memory [57]. The potential of these microbial-derived AhR ligands to impact brain development and function warrants further investigation. What is important in the context of this study is that alterations in the gut microbiome influenced by HMOs, for example, may influence different brain processes via the regulation of these microbial-associated metabolites.

Further, HICA has been shown to be produced by lactic acid bacteria [58] and was recently shown to exert both antimicrobial [59,60,61] and anti-inflammatory activity [62]. Another metabolite that was strongly increased, especially for adults and after IN, 2′FL, LNnT, BL2, and BL3 treatments, is the GABA. In line with recent studies [11,12], GABA correlated positively with *B. adolescentis*. GABA is formed via decarboxylation of glutamic acid [63] and extensive literature supports the link between altered GABAergic neurotransmission and numerous central and enteric nervous system disorders, such as behavioral or sleep alterations, pain, depression, changes in intestinal motility, gastric emptying, nociception, and acid secretion [64]. Further, GABA production for 3′SL and 6′SL is related to *Phocaeicola vulgatus*, reported as the second most potent GABA producer [64]. Finally, melatonin production markedly increased upon treatment with 2′FL, LNnT, BL2, and BL3 for children. As reviewed by Bubenik et al. (2002) [65], melatonin may have a direct effect on many gastrointestinal tissues but may also influence the digestive tract indirectly, via the central nervous system and the sympathetic and parasympathetic nerves. Melatonin prevents ulcerations of gastrointestinal mucosa by an antioxidant action, reduction in secretion of hydrochloric acid, stimulation of the immune system, fostering epithelial regeneration, and increasing microcirculation. Because of its unique properties, Bubenik et al. stated that melatonin could be considered for prevention or treatment of colorectal cancer, ulcerative colitis, gastric ulcers, irritable bowel syndrome, and childhood colic. While the stimulation of this spectrum of health-related metabolites further stresses the beneficial effects that could follow from HMO consumption, the results also indicate that different HMOs could exert complementary effects, given that 2′FL and sialylated HMOs seem to boost GABA production via different microbial species. 

Another remarkable finding was that 2′FL and LNnT exerted consistent bifidogenic effects across both age groups, while also increasing the butyrate-producing *A. hallii* (particularly LNnT that most strongly increased butyrate levels). LNnT also specifically increased *R. gnavus*, a gut commensal with the ability to degrade human mucin-glycans and HMOs [66]. Interestingly, the stimulation of these specific taxa was in line with the findings of recent human clinical trials. Indeed, administration of doses between 5–20 g/d of 2′FL, LNnT, and a mix thereof to healthy adults specifically increased *Bifidobacteriaceae* levels (mostly *B. adolescentis*) [22]. Further, upon dosing 10 g/d of a 4/1 mixture of 2′FL/LNnT to IBS patients, patients classified as responders (i.e., showing increase in *Bifidobacteriaceae*) had increased levels of *A. hallii* in their fecal microbiota [24]. This not only stresses the consistent effect of 2′FL and LNnT on the human gut microbiota across studies but it also demonstrates that microbial community changes observed with the SIFR^®^ technology, within as short as 24 h, are highly predictive for the microbial community composition changes observed in vivo upon repeated intake over multiple weeks. 

An overall treatment effect on microbial composition was that HMO supplementation resulted in higher α-diversity of the adult microbiota compared to fructans (as assessed with the reciprocal Simpson diversity index that accounts for both species richness and evenness). For children, mainly LNnT and BL1, maintained higher α-diversity. As a remark, the diversity in terms of species evenness tended to be lower compared to the untreated NSC. While this seems undesirable, it is evident when a substrate fulfills the prebiotic definition, i.e., when it is selectively stimulating the growth of specific microbes, it becomes highly abundant and renders the community less even. This has been demonstrated before for various prebiotic substrates [67], even in the presence of marked health benefits [68]. Nevertheless, a high microbial diversity is considered as a marker of a healthy microbiota, contributing to ecosystem resilience after disturbance of the microbiota, whereas a lower diversity and richness have been proposed as indicators of a wide array of pathological conditions, such as a metabolically unhealthy status in children with obesity [69], autism spectrum disorder, metabolic syndrome, or inflammatory bowel disease [70]. In this perspective, the higher overall diversity for HMO treatments is of great interest, particularly when HMOs would be used as a prebiotic by individuals who are at risk of microbial dysbiosis, such as elderly people undergoing broad-spectrum antibiotic treatment or people suffering from gastrointestinal disorders. 

Sialylated HMOs account for approximately 13% of the total HMOs in human milk and have multiple functions related to host health (reviewed in [71]). In fact, 6′SL was the treatment affecting the highest number of species from different families in children’s samples. Remarkably, 6′SL increased health-related *F. prausnitzii*, *R. torques*, *A. hallii* and *C. comes*. The strong effect of 6′SL on children’s gut microbiota could be explained by cross-feeding mechanisms, with primary degraders (i.e., bifidobacteria, among others) releasing HMOs degradants to be used by other species. This sharing mechanism has been reported for *B. bifidum* and other bifidobacterial communities [72], but also bifidobacteria and butyrate-producing bacteria [53]. 

Intriguingly, 3′SL supplementation increased folic acid levels in adults. The ability to produce folate has been reported for lactobacilli and bifidobacteria with different yields, and colonic folate absorption has also been proven [73]. Epidemiological studies have associated folate deficiency with an increased risk of breast cancer and shown that low folate homeostasis may induce hypomethylation of DNA, thereby promoting cancer in the proliferating cells of the colorectal mucosa [74,75]. Furthermore, increased folate intake is recommended for pregnant woman to support growth and development of the fetus [76]. Moreover, increased folate intake is also recommended for patients with inflammatory bowel diseases, contributing to the overall regulation of rectal cell turnover [77], further substantiating the unique impact of 3′SL on the gut microbiota.

The experimental design used in this research incorporated six donors in a high-reproducible SIFR^®^ technology, allowing for correlations that build further hypotheses on key species, driving certain pathways from specific treatments. Besides the marked correlations between e.g., acetate and *Bifidobacterium* species (in both age groups) and *Anaerobutyricum hallii* and butyrate (in adults), a series of other correlations were established. In line with the metabolic capability of the following species, butyrate production for children, mostly stimulated by fructans, correlated with the presence of *Coprococcus comes* [78], whereas the strong propionate production upon 6′SL treatment was linked to *Bacteroides fragilis* in children and *Phocaeicola massiliensis* in adults [4,79]. This demonstrates the potential of the SIFR technology to provide insights into species responsible for driving the production of specific metabolites within a complex pool of metabolites produced by potentially hundreds of microbial species.

A distinct advantage of the SIFR^®^ technology is that the absence of a host component (and thus the absence of, e.g., absorption of postbiotics or removal of gasses) enables unique insights in metabolite production and microbial composition that are hard to obtain in vivo. However, this also means that findings of ex vivo studies should be considered as complementary to clinical studies, rather than as potential replacements thereof. Despite the high predictivity of the SIFR^®^ technology for clinical findings [49], clinical studies are required as final proof of health benefits for the host.

## 5. Conclusions

Overall, age-dependent differences in microbiota composition greatly impacted prebiotic outcomes, thus advocating for the development of age-specific nutritional supplements. Following the concept of ‘targeted prebiotics’, defined as microbiota-directed fiber with a discrete structure [80], the current study provides mechanistic insights into the modulation of gut microbiota structure and function via HMOs supplementation, including metabolites beyond short-chain fatty acids, such as neurotransmitters or immune modulatory compounds. In this perspective, HMOs were shown to be promising modulators of the gut microbiota of adults and particularly children. Finally, given the specific effects of the individual HMOs on specific *Bifidobacterium* species (with marked differences being noted for 2′FL/LNnT compared to sialylated HMOs), mixing HMOs could result in more potent bifidogenic effects within a given donor, by providing a broader range of substrates with some selectively increasing targeted *Bifidobacterium* species. This was also confirmed during the current study where HMO blends were shown to exert potent bifidogenic effects. Such an approach could increase the rate of responders, not only in terms of bifidogenic effects, but also in terms of the production of health-related metabolites related to immune health and the gut-brain axis. This could particularly benefit human subjects that might be deficient in *Bifidobacterium* species (i.e., elderly people, IBS patients or people that underwent antibiotic treatment). 

## Data Availability

The datasets generated during and/or analyzed during the current study are available from the corresponding author upon reasonable request.

## Supplementary Materials

The following supporting information can be downloaded at: https://www.mdpi.com/article/10.3390/nu15071701/s1, Figure S1: Marked metabolite production occurred between 0–24 h, while the QC samples markedly co-localized. Figure S2: Fecal microbiota composition of children (6-year-old) and adults was fundamentally different. Figure S3: Kinetic sampling covered saccharolytic (0–24 h) and proteolytic fermentation processes (24–48 h). Figure S4: Given the marked increase in cell densities between test conditions, it was critical to convert proportional outcomes of shotgun sequencing to absolute levels. Figure S5: Impact on SCFA production when products were supplemented to the children’s and adult gut microbiota. Figure S6: HMOs exerted a remarkable bifidogenic effect for children, in contrast to fructans. For adults, fructans, 2′FL and LNnT exerted marked bifidogenic effects, which contrasted with the sialylated HMOs. Figure S7: Regularized Canonical Correlation Analysis (rCCA) to highlight correlations between microbial activity and composition for children. Figure S8: Regularized Canonical Correlation Analysis (rCCA) to highlight correlations between microbial activity and composition for adults.
