# Supplementary material for: HMOs Exert Marked Bifidogenic Effects on Children’s Gut Microbiota Ex Vivo, Due to Age-Related Bifidobacterium Species Composition
Source: Nutrients. 2023 Mar 30;15(7):1701. doi: 10.3390/nu15071701 (PMC10097135; doi:10.3390/nu15071701)
Supplement: Supplementary file 1 [file nutrients-15-01701-s001.zip › nutrients-2216692-supplementary.pdf]

nutrients
MDPI

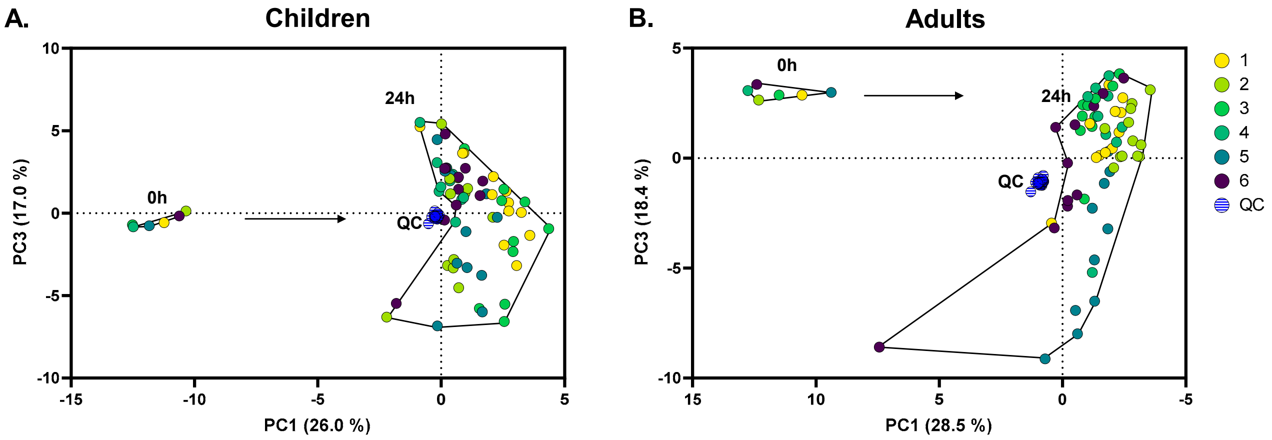

Figure S1. Marked metabolite production occurred between 0-24h, while the QC samples markedly co-localized. Principal component analysis (PCA) of level 1-annotated metabolites (LC-MS) for children (A) and adults (B) (n=6), both at 0h and 24h of treatment with single HMOs, blends thereof and reference prebiotics. QC= quality control sample (= pooled sample of all samples). QC = quality control; HMO = human milk oligosaccharide; LC-MS = Liquid chromatography coupled with mass spectrometry.

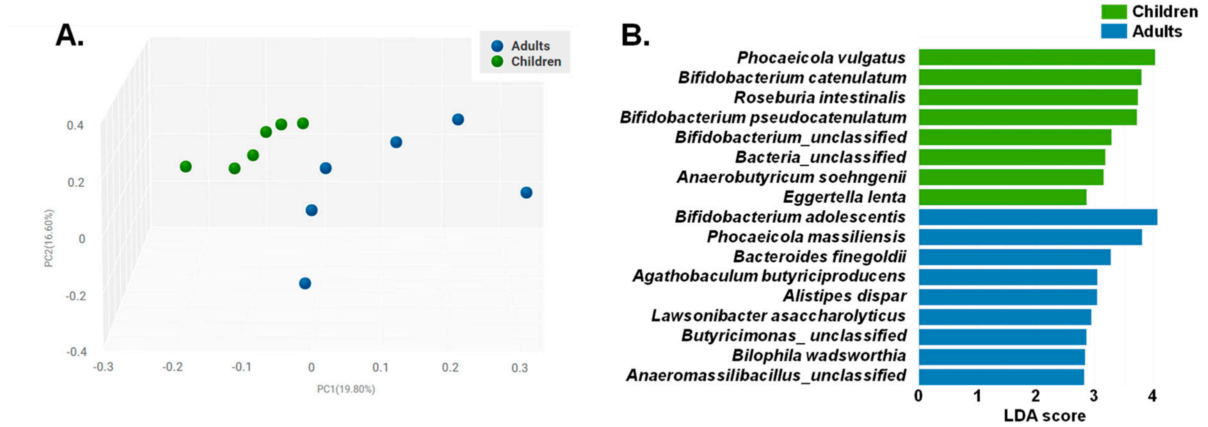

Figure S2. Fecal microbiota composition of children (6 years old) and adults was fundamentally different. (A) Principal coordinates analysis (PCoA) based on Bray-Curtis distance for microbial species (%), as quantified via shallow shotgun sequencing (p = 0.024). (B) Linear discriminant analysis effect size (LEfSe) at species level shows the taxa most likely to explain differences between children and adults (LDA threshold = 2).

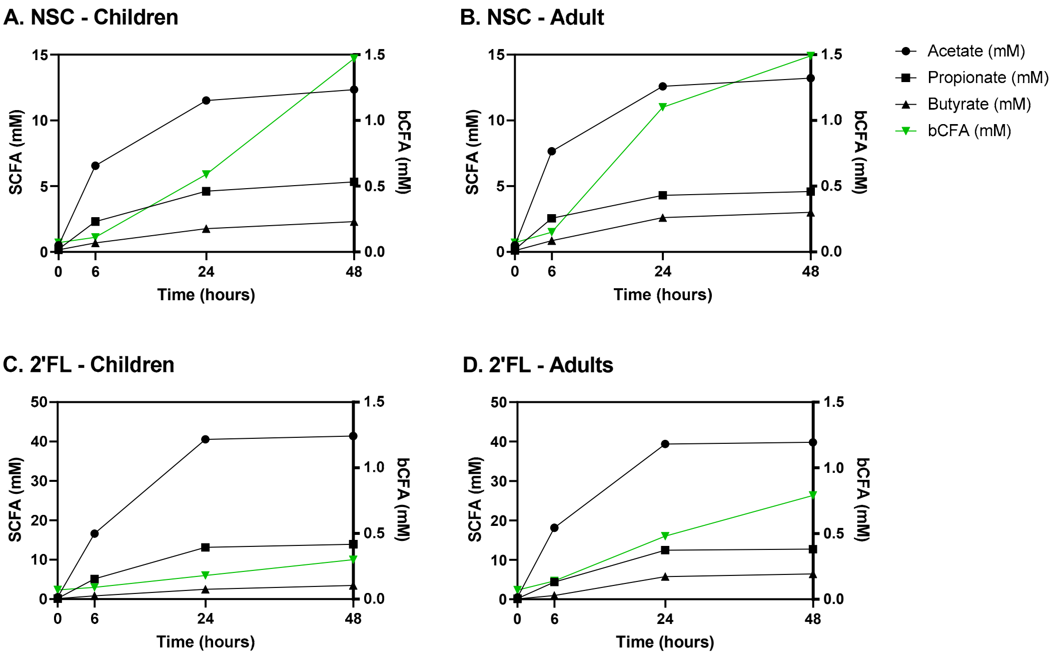

Figure S3. Kinetic sampling covered saccharolytic (0-24h) and proteolytic fermentation processes (24-48h). SCFA and bCFA levels (mM) in the no substrate control (NSC) (A/) and upon 2′FL treatment (C, D), averaged across 6 children (A/C) or 6 human adults (B/D). SCFA = short chain fatty acids; bCFA = branched fatty acids; NSC = no substrate control; 2′FL = 2′Fucosyllactose.

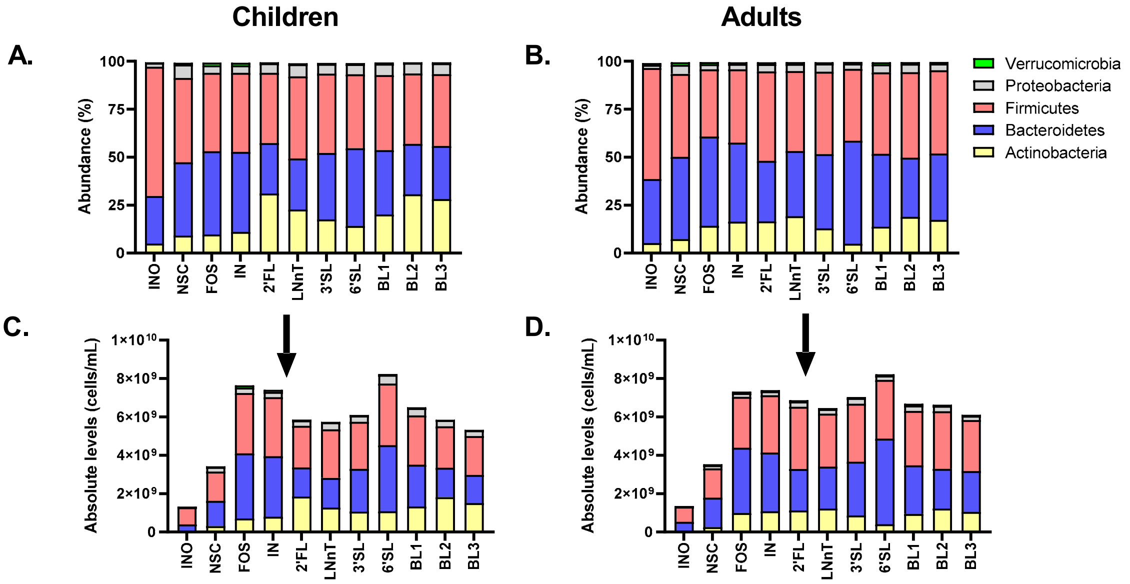

Figure S4. Given the marked increase in cell densities between test conditions, it was critical to convert proportional outcomes of shotgun sequencing to absolute levels. Impact of single HMOs (2′FL, LNnT, 3′SL, 6′SL), mixtures thereof (BL1, BL2, BL3) and reference prebiotics (FOS and IN) on microbial composition at phylum level, both in terms of relative (%) (A, B) and absolute levels (cells/mL) (C-D) for the gut microbiota of 6 years old children and human adults (n=6), upon 24h of initiation of the treatment, compared to a no substrate control (NSC), as tested with the *ex vivo* SIFR® technology. 2′FL = 2′Fucosyllactose; LNnT = Lacto-N-neotetraose; 3′SL = 3′Sialyllactose; 6′SL = 6′Sialyllactose; BL1/2/3 = HMO Blend 1/2/3; IN = inulin; FOS = fructooligosaccharides.

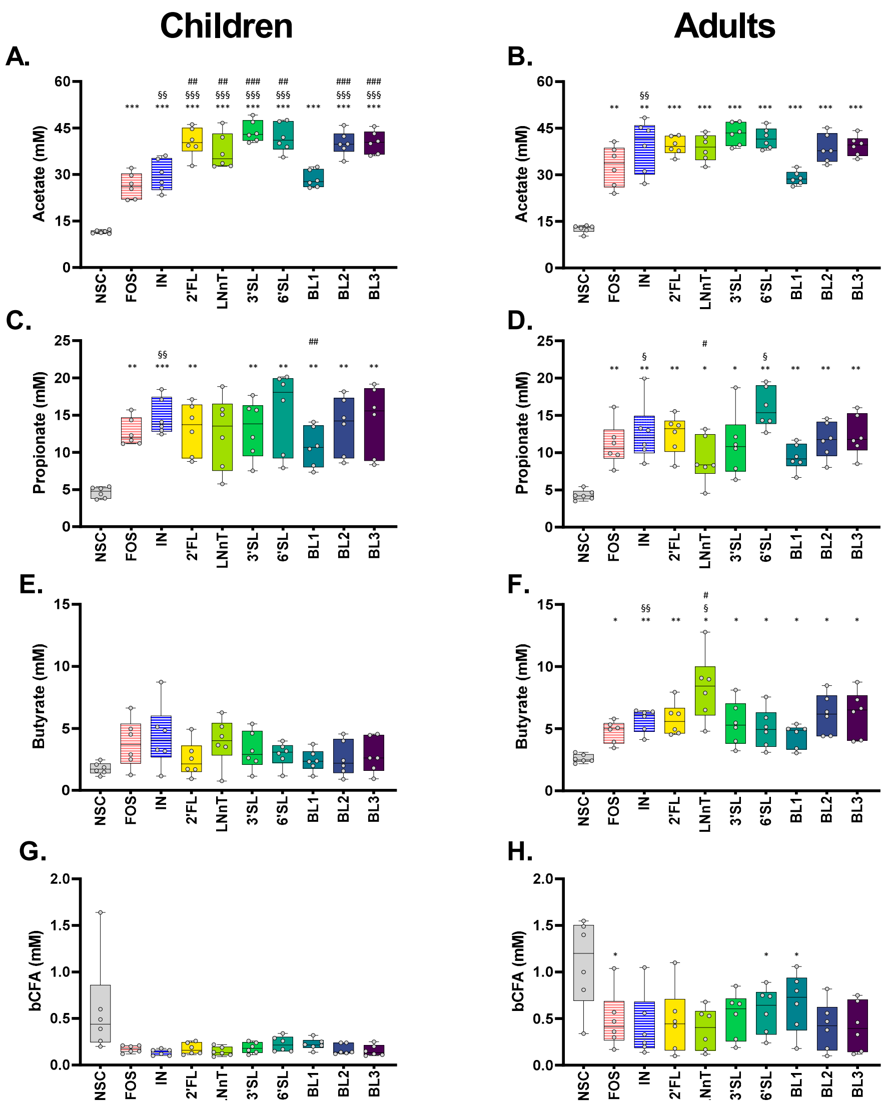

Figure S5. When administered to the children's microbiota, all treatments significantly increased acetate and propionate, with remarkable effects of HMOs on acetate. When administered to the adult microbiota, all treatments significantly increased acetate, propionate and butyrate, with remarkable effects of 6'SL and LNnT on propionate and butyrate, respectively. Impact of single HMOs (2'FL, LNnT, 3'SL, 6'SL), mixtures thereof (BL1, BL2, BL3) and reference prebiotics (FOS and IN) on acetate (A, B), propionate (C, D), butyrate (E, F), and bCFA (G, H) levels for simulated gut microbiota of children (A, C, E, G; n=6) or adults (B, D, F, H; n=6), at 24h upon initiation of treatment, compared to a no substrate control (NSC), as tested with the *ex vivo* SIFR® technology. Statistical differences between treatments and NSC are indicated with asterisks [* ($p_{adjusted} < 0.05$), ** ($p_{adjusted} < 0.01$) or *** ($p_{adjusted} < 0.001$)]. Further, statistical differences between single HMOs/HMO blends and FOS are indicated with §/§§/§§§, while differences with IN are indicated with #/##/###. 2'FL = 2'Fucosyllactose; LNnT = Lacto-N-neotetraose; 3'SL = 3'Sialyllactose; 6'SL = 6'Sialyllactose; BL1/2/3 = HMO Blend 1/2/3; IN = inulin; FOS = fructooligosaccharides; bCFA = branched fatty acids.

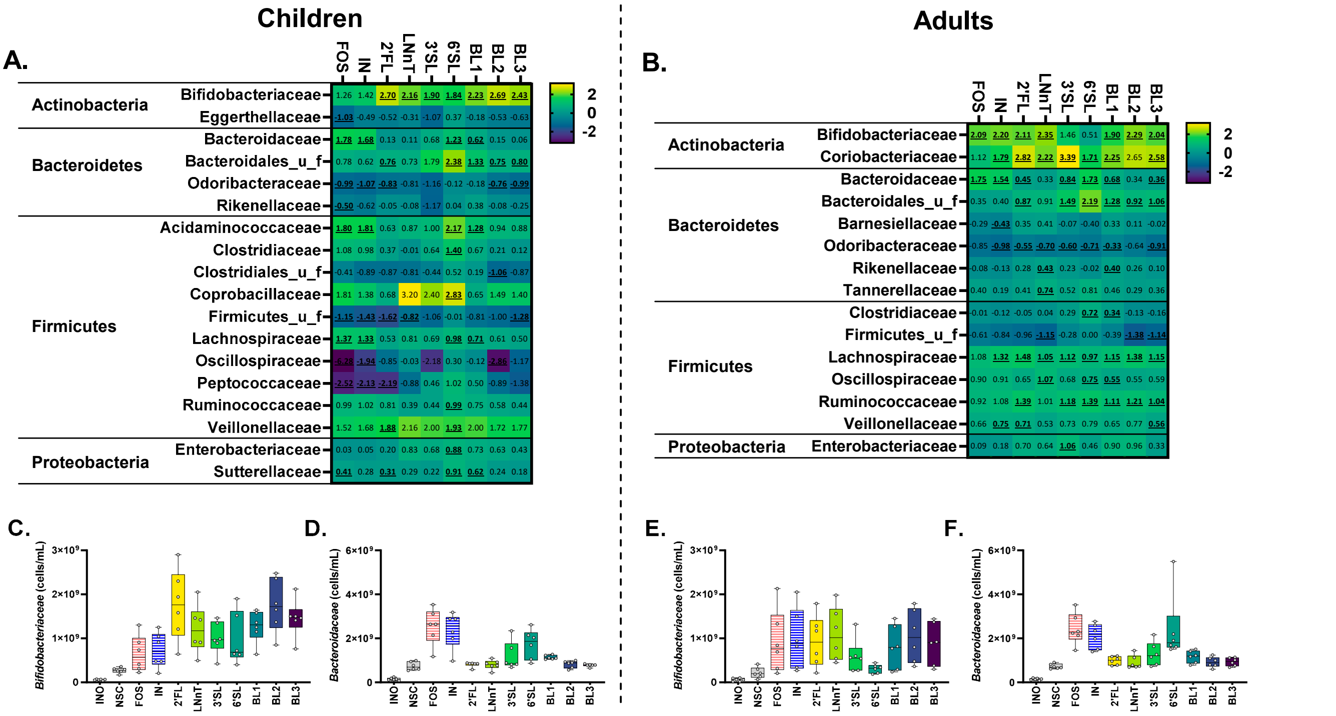

Figure S6. HMOs exerted a remarkable bifidogenic effect for children, in contrast to fructans. For adults, fructans, 2′FL and LNnT exerted marked bifidogenic effects, which contrasted with the sialylated HMOs. Impact of single HMOs (2′FL, LNnT, 3′SL, 6′SL), mixtures thereof (BL1, BL2, BL3) and reference prebiotics (FOS and IN) on microbial composition at family level for children (A, C, D) and adults (B, E, F) (n=6), at 24h upon initiation of treatment, as tested with the *ex vivo* SIFR® technology. Heatmaps represent average values of microbial taxa (n=6 per age group) that were significantly affected by any of the treatments (FDR = 0.10), expressed as log2(ratio treatment *vs.* NSC), for children (A) and adults (B). Significant differences are indicated by bold and underlining. (C-F) Box plots representing the abundances (cells/mL) of *Bifidobaceriaceae* (C, E) and *Bacteroidaceae* (E, F). NSC = no substrate control; 2′FL = 2′Fucosyllactose; LNnT = Lacto-N-neotetraose; 3′SL = 3′Sialyllactose; 6′SL = 6′Sialyllactose; BL1/2/3 = HMO Blend 1/2/3; IN = inulin; FOS = fructooligosaccharides.

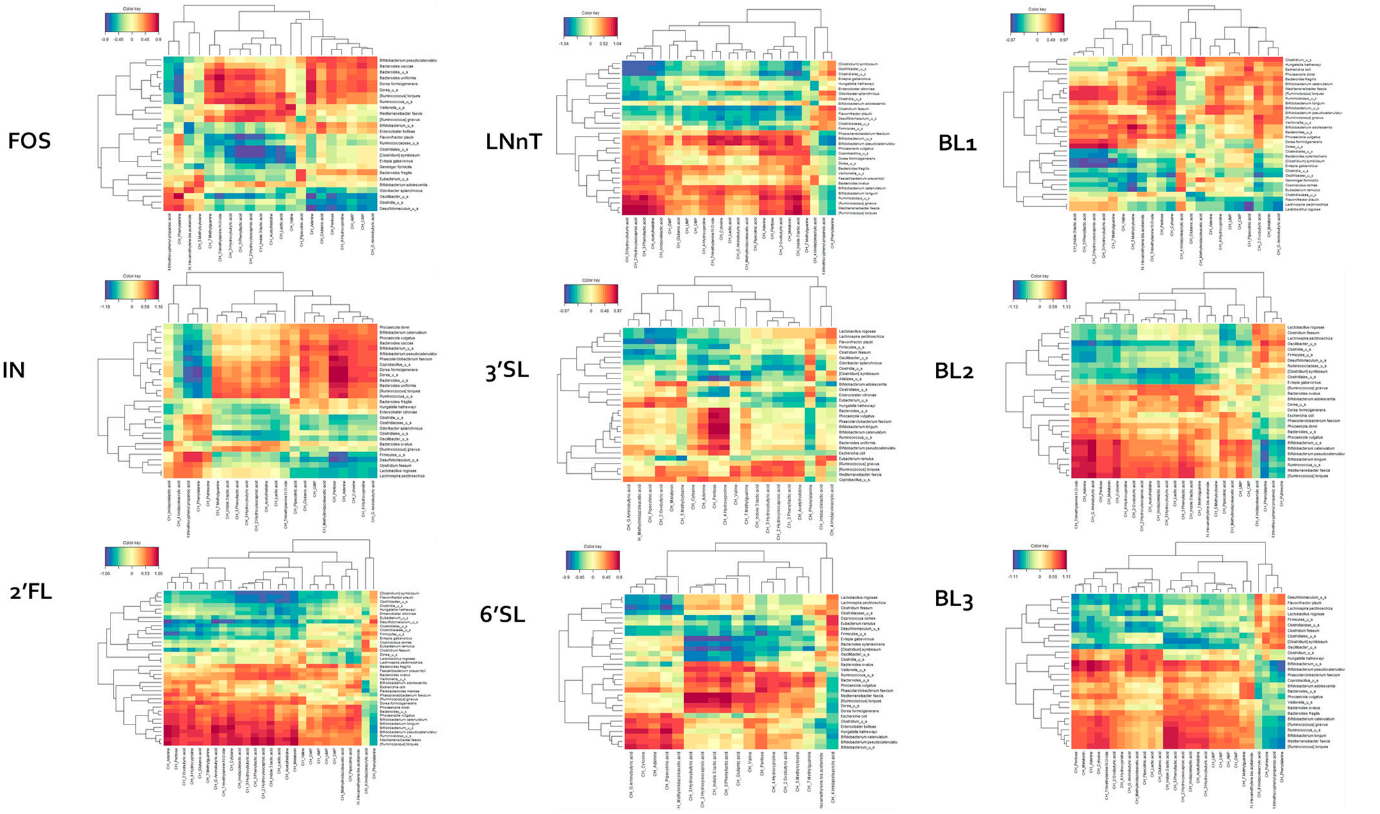

Figure S7. Regularized Canonical Correlation Analysis (rCCA) to highlight correlations between microbial activity and composition for single HMOs (2'FL, LNnT, 3'SL, 6'SL), mixtures thereof (BL1, BL2, BL3) and reference prebiotics (FOS and IN) at 24h upon initiation of treatment, as tested with the *ex vivo* SIFR® technology for children (n=6). More specifically, the correlation was established between significantly affected species and selected metabolites reported in Figure 6A, with a cut-off of 0.65. In order to make the correlation, also NSC samples were included. NSC = no substrate control; 2'FL = 2'Fucosyllactose; LNnT = Lacto-N-neotetraose; 3'SL = 3'Sialyllactose; 6'SL = 6'Sialyllactose; BL1/2/3 = HMO Blend 1/2/3; IN = inulin; FOS = fructooligosaccharides.

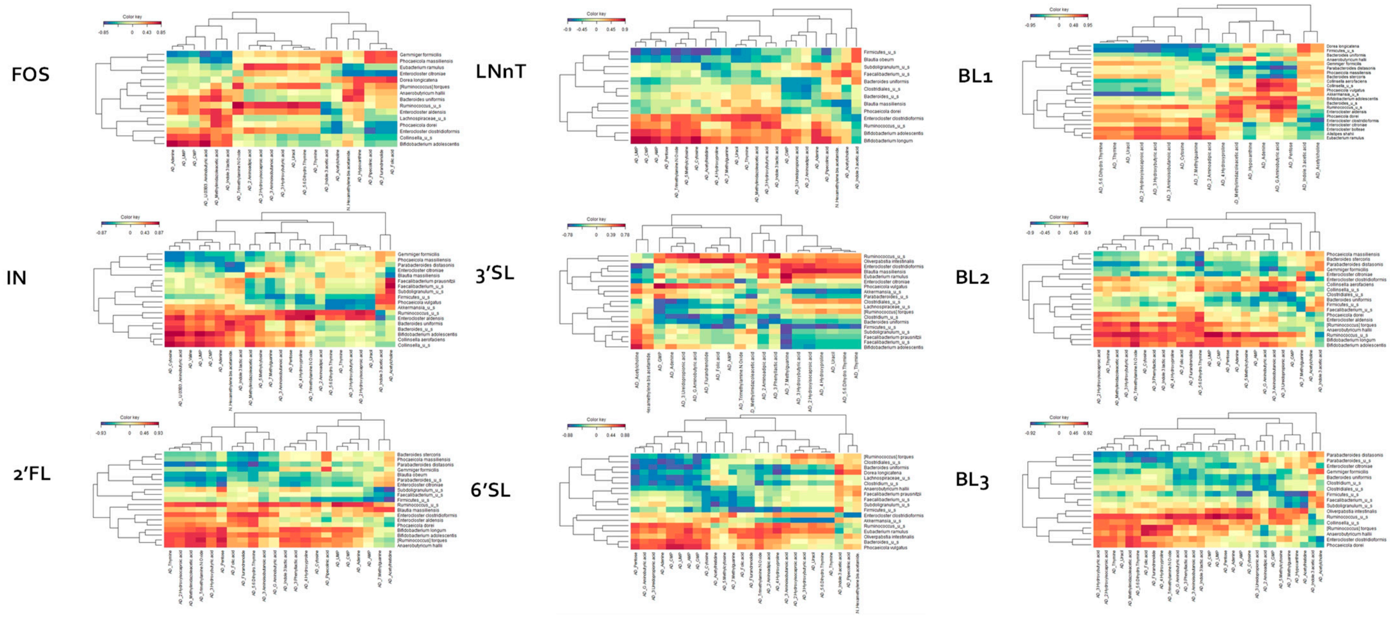

Figure S8. Regularized Canonical Correlation Analysis (rCCA) to highlight correlations between microbial activity and composition for single HMOs (2'FL, LNnT, 3'SL, 6'SL), mixtures thereof (BL1, BL2, BL3) and reference prebiotics (FOS and IN) at 24h upon initiation of treatment, as tested with

the *ex vivo* SIFR® technology for adults (n=6). More specifically, the correlation was established between significantly affected species and selected metabolites reported in Figure 6B, with a cut-off of 0.65. In order to make the correlation, also NSC samples were included**.** NSC = no substrate control; 2’FL = 2’Fucosyllactose; LNnT = Lacto-N-neotetraose; 3’SL = 3’Sialyllactose; 6’SL = 6’Sialyllactose; BL1/2/3 = HMO Blend 1/2/3; IN = inulin; FOS = fructooligosaccharides.
